# Youths Experiencing Parental Death Due to Cancer

**DOI:** 10.1001/jamanetworkopen.2025.19106

**Published:** 2025-07-07

**Authors:** Alexandra L. Potter, Benjamin-Samuel Schlüter, Monica J. Alexander, Chi-Fu Jeffrey Yang, Mathew V. Kiang

**Affiliations:** 1 Division of Thoracic Surgery, Department of Surgery, Massachusetts General Hospital, Boston; 2 Max Planck Institute for Demographic Research, Rostock, Germany; 3 Department of Statistical Sciences, University of Toronto, Toronto, Canada; 4 Department of Sociology, University of Toronto, Toronto, Canada; 5 Department of Epidemiology and Population Health, Stanford University, Stanford, California

## Abstract

This cross-sectional study estimates the number of youths who experienced parental death due to cancer annually overall and by race and ethnicity from 1999 to 2020.

## Introduction

The death of a parent from cancer is associated with increased risks of self-injury^1^ and short- and long-term adverse psychological outcomes^2^ among children. However, the number of children who lose 1 or more parents to cancer each year in the US is unknown. The objectives of this study were to estimate the number of youths (aged <18 years) who lose 1 or more parents to cancer overall and by race and ethnicity in the US and to assess changes in the incidence of parental cancer death from 1999 to 2020.

## Methods

This repeated cross-sectional retrospective study used several publicly available data sources, including population estimates from the US Census Bureau, male fertility rates from the Human Fertility Collection, and both female fertility rates and mortality data from the National Center for Health Statistics (eMethods 1 in Supplement 1). The Stanford University institutional review board deemed this study exempt from review and informed consent because it used publicly available de identified data on deceased individuals. This study followed the Strengthening the Reporting of Observational Studies in Epidemiology (STROBE) reporting guideline.

For this study, we adapted a demographic matrix projection model to estimate the number of youths who experience parental cancer death and the incidence of parental cancer death per 1000 youths overall and by race and ethnicity in the US each year from 1999 to 2020. Data on race and ethnicity, which were primarily self-reported, were identified in the data sources (eMethods 2 in Supplement 1). The model and its assumptions are summarized in eMethods 3 in Supplement 1 and are described in detail in our prior publication.^3^ We included deaths from any malignant neoplasm (eAppendix in Supplement 1). Bootstrap CIs were estimated using 2000 bootstrap samples. Data were analyzed using R software version 4.1.3 (R Project for Statistical Computing) from November 4, 2024, to April 19, 2025.

## Results

Between 1999 and 2020, there were 12 644 868 deaths due to cancer, with a median (IQR) age of 72 (62-81) years. Most deaths occurred among males (6 608 436 deaths [52.3%]); 5.7% of deaths were among Hispanic individuals, 11.4% were among non-Hispanic Black individuals, and 80.1% were among non-Hispanic White individuals.

The modeling analysis estimated that 1 350 000 (95% CI, 1 303 000-1 398 000) youths lost 1 or more parents to cancer in the US from 1999 to 2020. The median (IQR) age at the time of parental cancer death was 13 (8-15) years; 433 009 youths (36.1%) were aged 10 years or younger at time of parental cancer death.

**Figure 1.**
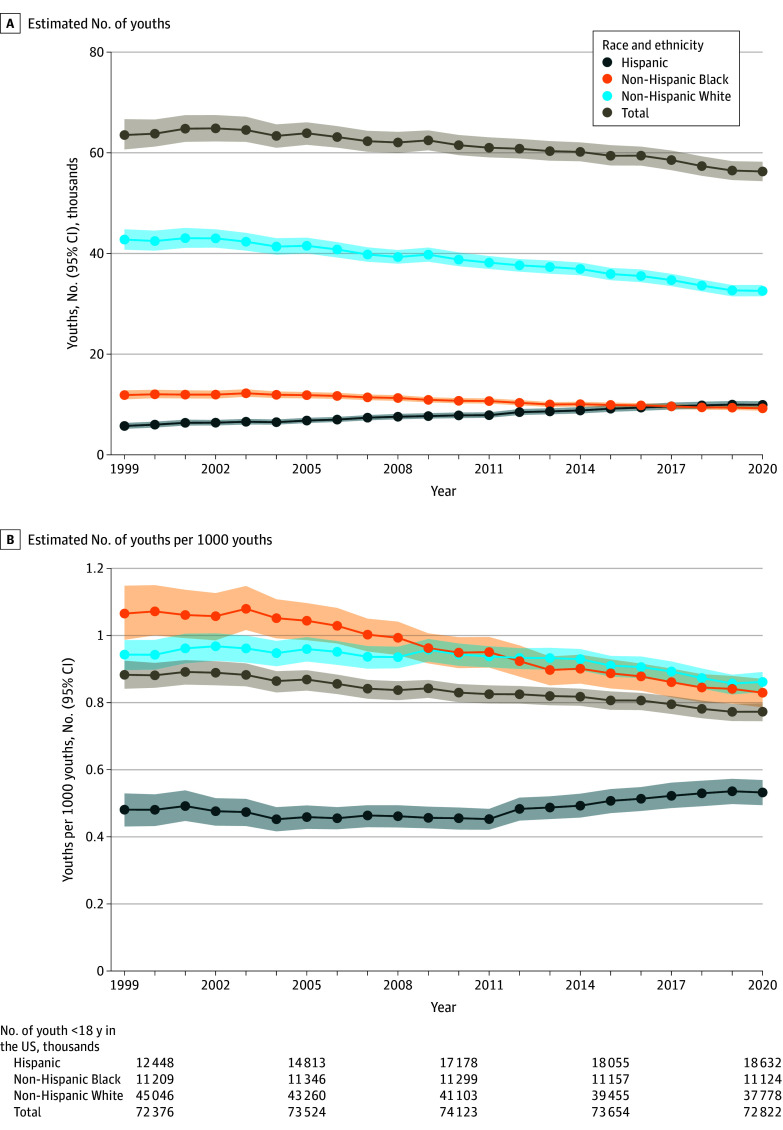
Youths Who Lost 1 or More Parents to Cancer in the US Numbers in the risk table (B) indicate the total number of youths (in thousands) living in the US each year. These numbers provide information about the number of youth at risk of experiencing parental cancer mortality each year. These numbers do not indicate the number of youths who experienced parental cancer death each year.

In 2020, an estimated 56 300 (95% CI, 54 400-58 200) youths experienced parental cancer death, representing an incidence of 0.77 (95% CI, 0.75-0.80) youths experiencing parental cancer death per 1000 youths (Figure 1). An estimated 25 500 (95% CI, 24 400-26 600) youths lost a mother to cancer and an estimated 30 400 (95% CI, 28 900-31 900) youths lost a father to cancer in 2020. Subgroup analyses by race and ethnicity are shown in Figure 1.

**Figure 2.**
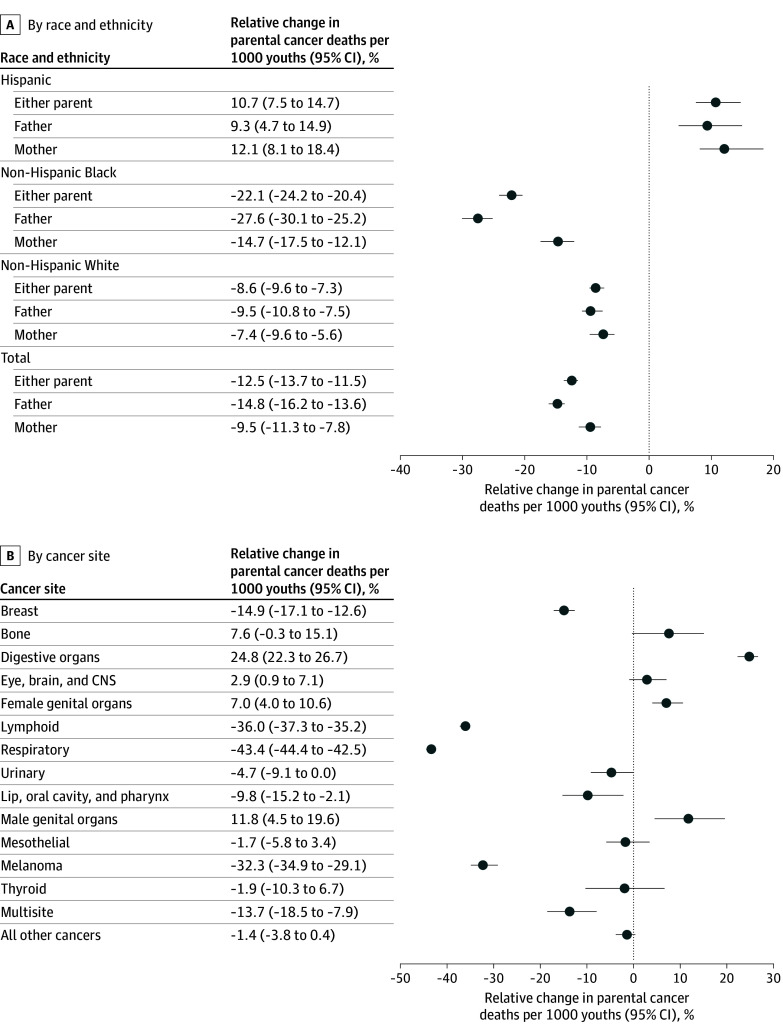
Change in the Number of Youth Who Lost 1 or More Parents to Cancer Per 1000 Youth in 1999 vs 2020 Bone includes bone and articular cartilage; lymphoid, lymphoid, hematopoietic, and related tissue; respiratory, respiratory and intrathoracic organs; urinary, urinary tract (kidney, ureter, bladder); mesothelial, mesothelial and soft tissue; melanoma, melanoma and other malignant neoplasms of the skin; and thyroid, thyroid and other endocrine glands. The *International Statistical Classification of Diseases and Related Health Problems, Tenth Revision* (*ICD-10*) cause of death codes for each cancer site are shown in the eTable in Supplement 1.

From 1999 to 2020, the incidence of parental cancer death declined by −12% (95% CI, −14% to −11%), which was driven by a larger reduction in paternal deaths (change, −15% [95% CI, −16% to −14%]) compared with maternal deaths (change, −9% [95% CI, −11% to −8%]). (Figure 2A). In a subgroup analysis by race and ethnicity, from 1999 to 2020, the incidence of parental cancer death decreased among non-Hispanic Black youths and non-Hispanic White youths, while it increased among Hispanic youths. The incidence of parental cancer death was either stable or declined from 1999 to 2020 among all cancer sites except for cancers of the digestive system and male and female reproductive organ systems (Figure 2B).

## Discussion

In this cross-sectional modeling study, an estimated 56 300 youth lost 1 or more parents to cancer in the US in 2020, at a median age of 13 years. In the last 20 years, estimated parental cancer death declined among non-Hispanic populations but increased among Hispanic youth. These findings are corroborated by prior studies reporting declines in cancer mortality among younger White and Black adults and increases in cancer mortality among younger Hispanic adults.^4,5^ The data highlight that parental loss due to cancer among youths is a notable public health issue in the US and emphasize the importance of programs to support youth who experience parental cancer death.

Limitations of this study include the model’s assumption of equivalent fertility rates among individuals who die from cancer and those who do not as well as limited data available for male fertility rates. Additionally, the COVID-19 pandemic may have impacted estimated parental cancer death in 2020, as delays in timely diagnosis and treatment, combined with elevated mortality risk due to infection,^6^ could have contributed to worse outcomes for parents with cancer.

## Notes

Supplement 1. **eMethods 1.** Details of the Data Sources **eMethods 2.** Details About the Use of Race and Ethnicity in the Analysis **eMethods 3.** Tabulating Death Counts, Summary of the Matrix Kinship Model, and Model Assumptions **eAppendix.** Reproducible Code and Data **eTable.** Defining Causes of Death Due to Cancer
eReferences.

Supplement 2.
Data Sharing Statement
