# Supplementary material for: Youths Experiencing Parental Death Due to Cancer
Source: JAMA Netw Open. 2025 Jul 7;8(7):e2519106. doi: 10.1001/jamanetworkopen.2025.19106 (PMC12235492; doi:10.1001/jamanetworkopen.2025.19106)
Supplement: Supplement 1. — eMethods 1. Details of the Data Sources eMethods 2. Details About the Use of Race and Ethnicity in the Analysis eMethods 3. Tabulating Death Counts, Summary of the Matrix Kinship Model, and Model Assumptions eAppendix. Reproducible Code and Data eTable. Defining Causes of Death Due to Cancer eReferences. [file jamanetwopen-e2519106-s001.pdf]

Supplemental Online Content

Potter AL, Schlüter B, Alexander MJ, Yang CJ, Kiang MV. Youths experiencing parental death due to cancer. *JAMA Netw Open*. 2025;8(7):e2519106.
doi:10.1001/jamanetworkopen.2025.19106

eMethods 1. Details of the Data Sources

eMethods 2. Details About the Use of Race and Ethnicity in the Analysis

eMethods 3. Tabulating Death Counts, Summary of the Matrix Kinship Model, and Model Assumptions

eAppendix. Reproducible Code and Data

eTable. Defining Causes of Death Due to Cancer

eReferences.

This supplemental material has been provided by the authors to give readers additional information about their work.

© 2025 Potter AL et al. *JAMA Network Open*.

eMethods 1. Details of the Data Sources

This modeling analysis did not require review by the Stanford University Institutional Review Board, as it does not meet the criteria for human subjects research. Specifically, this analysis uses publicly available, secondary, deidentified data with no risk of reidentification. Here, we provide more information about the data sources used in this modeling analysis.

Data Sources

In order to estimate parental death by cause of death, we required (a) population estimates; (b) death counts by cause of death; and (c) fertility rates for both females and males from 1999 to 2020.

Population estimates. This modeling analysis used population estimates from the US Census Bureau for the period 1999 through 2020. These population estimates are stratified by age, race, ethnicity, and sex, and serve as the denominators for mortality and fertility rates.

Mortality data. We used the publicly available Multiple Cause-of-Death Records from the National Center for Health Statistics (NCHS).[1] These files include data from January 1999 through December 2020 and contain individual-level information for all deaths that occur in the US including decedents' cause of death, month and year of death, age, sex, race, and ethnicity.

Female fertility data. The NCHS provides publicly available Birth Files, which contain information for all births that occur in the US including mothers' age, race, and ethnicity. We used fertility data from 1999 through 2020 from the NCHS "Health, United States" series, which summarizes the Birth Files to calculate mothers' birth and fertility rates[2,3] by age, race, and ethnicity.

Male fertility data. Age-, race-, and ethnicity-specific fertility rates for males in the US are not available. We used male fertility for the total population from the Human Fertility Collection (HFC),[4] to model age-, race-, and ethnicity-specific fertility for males. A detailed description of how we estimated male fertility rates is included in our prior publication.[5]

© 2025 Potter AL et al. *JAMA Network Open*.

eMethods 2. Details About the Use of Race and Ethnicity in the Analysis

In the present analysis, we conducted a subgroup analysis where we evaluated parental cancer mortality stratified by race and ethnicity. The racial and ethnic groups we evaluated were Hispanic, non-Hispanic Black, and non-Hispanic White. These groupings were selected because they were consistent with the National Center for Health Statistics bridged-race categories,[6] were consistent across both the mortality and fertility data sets (described above), and had sufficient sample size when using the required one-year age groupings.

eMethods 3. Tabulating Death Counts, Summary of the Matrix Kinship Model, and Model Assumptions

Tabulating Death Counts

We tabulated death counts for (a) deaths due to any cancer site; (b) deaths due to specific cancer sites (see **eTable1** for the ICD-10 codes used to define death by cancer site); and (c) all other causes of death excluding deaths due to cancer. Death counts were generated for each of these causes of death groupings by racial/ethnic group and sex in one-year age bins (i.e., <1, 1, 2, …, 85+ years old). Deaths due to cancer and all other causes of death were tabulated from January 1st, 1999 to December 31st, 2020.

Summary of the Matrix Kinship Model

To estimate the number of youth that experienced parental cancer death, we used a previously developed two-sex time-variant kinship matrix model.[7-9] The model assumptions and limitations are described in detail in our prior publication[5] and are summarized below.

© 2025 Potter AL et al. *JAMA Network Open*.

Briefly, the model estimates, for every year, the number of living children (aged <18 years) an average individual has at every age in year t; these estimates are based on the race-, ethnicity-, age- and sex-specific survival probabilities of children up to age 18 and the race-, ethnicity-, age- and sex-specific fertility rates of the parents (data sources described above). The model then estimates the number of children experiencing parental death due to a given cause (e.g., cancer) by combining the estimates of the number of surviving children with the race-, ethnicity-, age-, and sex- cause-specific death counts. The primary outcomes of interest in the present study were the (1) model's estimates of the absolute number of children aged less than 18 years who lost one or more parents to cancer each year and (2) the model's estimates of the percentage of children aged less than 18 years who lost one or more parents to cancer each year. The sources of variation and incorporation of uncertainty into the model's estimates are described in our prior publication.[5]

Model Assumptions and Limitations

One key assumption of the model is that fertility among individuals who die from cancer is the same as fertility among individuals who die from other causes (i.e., individuals who die from cancer have the same number of children at the time of death as individuals who die from other causes). We discuss this assumption in detail below. Other key assumptions of the model, as well as model limitations, have been described in detail in our prior publication.[5]

To evaluate the validity of the assumption that fertility among individuals who die from cancer is the same as fertility among individuals who die from other causes, we investigated the age at which cancer deaths occurred relative to age-specific fertility rates. If we found that many cancer deaths occurred at ages when individuals experienced their peak fertility, these data would suggest that

© 2025 Potter AL et al. *JAMA Network Open*.

cancer would have a notable impact on individuals' fertility. Alternatively, if we found that many cancer deaths occurred at ages well after individuals experienced their peak fertility, these data would suggest that cancer would have minimal impact on individuals' fertility.

The cumulative proportion of cancer deaths by age versus age-specific fertility rates from 1999-2020 is shown in the figure below. From age 10 to 39, the cumulative proportion of cancer deaths was overall very low and increased gradually. From age 40 to 50, the increase in the cumulative proportion of cancer deaths accelerated. In contrast, the age-specific fertility rate peaked around age 30 and then declined sharply; by age 40, the age-specific fertility rate was more than 75% lower than the peak fertility rate. Importantly, most cancer deaths occurred among adults between 40-50, when the fertility rate was very low.

Cumulative Proportion of Deaths by Age and Age-Specific Fertility Rates Among Females and Males from 1999-2020. The lines indicate the cumulative proportion of deaths among males and female: the red line represents deaths due to cancer, the blue line represents deaths due to non-cancer causes, and the green line represents death due to any cause (including cancer or non-cancer causes). The histograms show the distribution of age-specific fertility rates among males and females.

© 2025 Potter AL et al. *JAMA Network Open*.

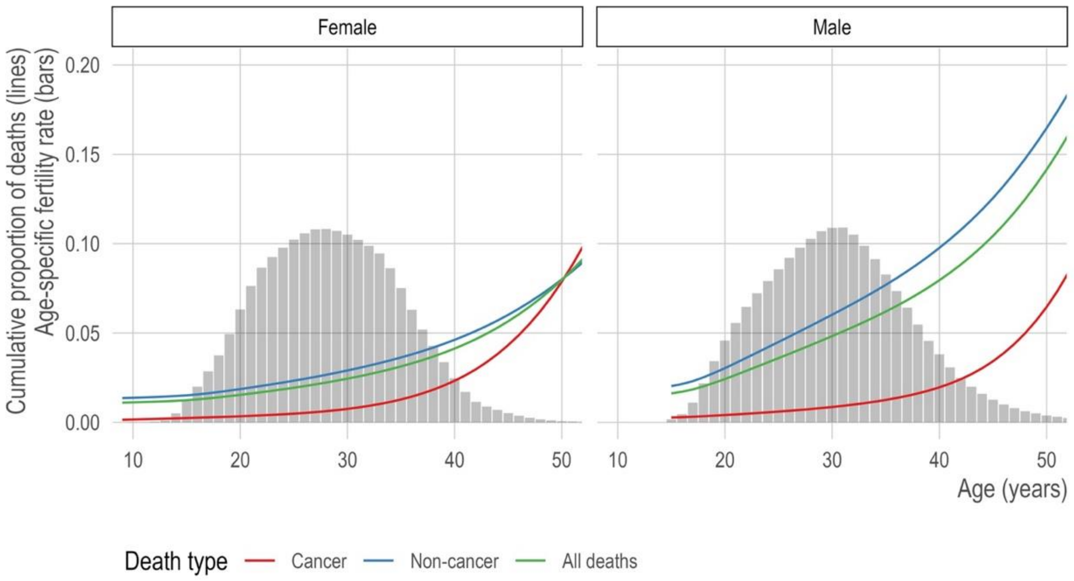

Next, we evaluated the proportionate remaining fertility by age from 1999-2020. By age 40, the vast majority of reproduction has been completed. For example, by age 40, only 3.1% of female fertility remains and 8.0% for males. Taken together, these data suggest that the impact of cancer deaths on fertility rates is likely minimal given that most cancer deaths occur well *after* reproductive age. However, there are limitations to this analysis. It should be noted that this analysis does not account for the fact that people who die from cancer may experience lower fertility during the time preceding cancer death (e.g., someone diagnosed with cancer may experience lower fertility during the period between cancer diagnosis and death). Additionally, in the U.S., male fertility rates decline more slowly with age when compared to female fertility rates.[10] As a result, it is likely that cancer deaths have a greater impact on male fertility compared to female fertility.

Proportionate Fertility Remaining by Age and Sex from 1999-2020. The lines indicate the total proportion of remaining fertility by age and sex aggregated over the entire period of interest (1999-2020).

© 2025 Potter AL et al. *JAMA Network Open*.

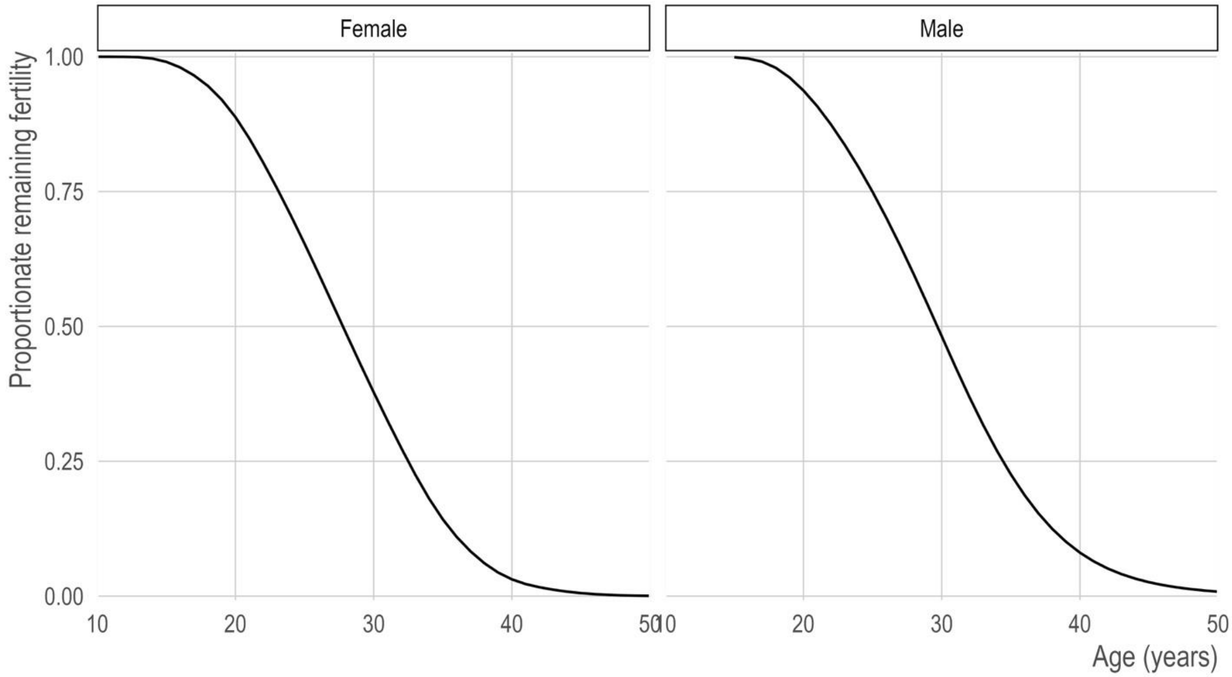

To assess the impact of this assumption on our results, we reran our simulation under different fertility scenarios. Specifically, we lowered the fertility rate of those who died of cancer to be 5%, 10%, 15%, and 20% lower than the fertility rate of those who did not die of cancer (our baseline model). The figure below shows the results of this sensitivity analysis. Under the most conservative scenario (i.e., 20% lower fertility), about 1.08 million youth (95% CI: 1.04, 1.12) experienced parental death due to cancer compared to our baseline scenario of 1.35 million youth. Of note, in this sensitivity analysis, we tested lower *overall* fertility rates (e.g., we lowered the fertility rate for individuals' entire reproductive period). It is unlikely, however, that individuals who die from cancer experience lower fertility during their entire reproductive period; the impact of cancer on fertility is likely isolated to a period of time (on the order of months to a few years) preceding cancer death. Thus, it is likely that the results of this sensitivity analysis are underestimates of the true number of youth experiencing parental mortality due to cancer.

© 2025 Potter AL et al. *JAMA Network Open*.

Results of Sensitivity Analysis to Changing Fertility Rate of Those Who Die of Cancer. The cumulative numbers of youth experiencing parental deaths due to cancer from 1999 – 2020 under different fertility scenarios are shown. The "Baseline model" (represented by the gray line) assumes that fertility among individuals who die of cancer is the same as fertility among individuals who die of other causes. In sensitivity analyses, we conducted the simulation under different fertility scenarios, examining the impact of lower fertility among those who died of cancer.

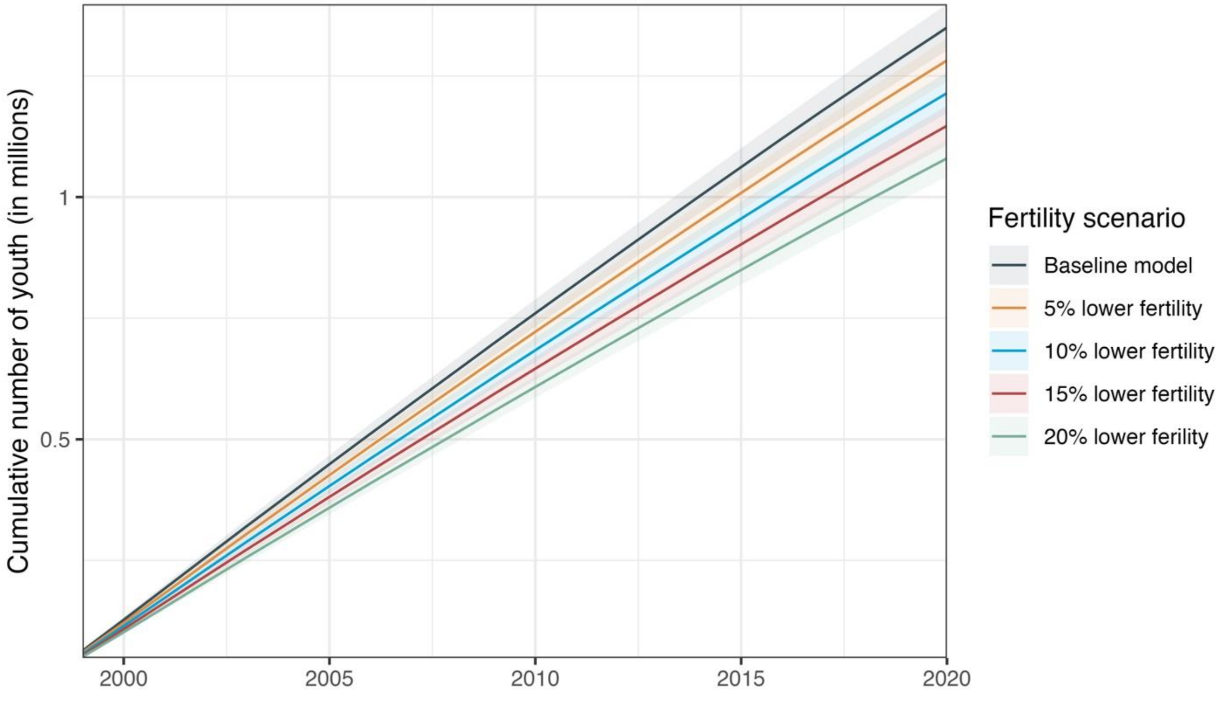

© 2025 Potter AL et al. *JAMA Network Open*.

eAppendix 1. Reproducible Code and Data. Reproducible code is available at: https://github.com/mkiang/parental_deaths_cancer. This online code repository includes the code necessary to download the publicly available multiple cause of death mortality files from the NCHS. Smaller files, such as the female fertility rates, also from the NCHS, are available directly from the repository. Lastly, for full reproducibility, large files generated from our analysis are available via the Open Science Framework (OSF) and instructions for downloading these files from OSF are available on the repository.

© 2025 Potter AL et al. *JAMA Network Open*.

eTable1: Defining Causes of Death Due to Cancer	
**Cancer Site**	**ICD-10 Codes**
Bone and Articular Cartilage	C40, C41
Breast	C50
Digestive Organs	C15, C16, C17, C18, C19, C20, C21, C22, C23, C24, C25, C26
Eye, Brain, and Other Parts of the CNS	C69, C70, C71, C72
Female Genital Organs	C51, C52, C53, C54, C55, C56, C57, C58
Lip, Oral Cavity, and Pharynx	C00, C01, C02, C03, C04, C05, C06, C07, C08, C09, C10, C11, C12, C13, C14
Lymphoid, Hematopoietic, and Related Tissue	C81, C82, C83, C84, C85, C86, C87, C88, C90, C91, C92, C93, C94, C95, C96
Male Genital Organs	C60, C61, C62, C63
Melanoma and Other Malignant Neoplasms of the Skin	C43, C44
Mesothelial and Soft Tissue	C45, C46, C47, C48, C49
Respiratory and Intrathoracic Organs	C30, C31, C32, C33, C34, C35, C36, C37, C38, C39
Thyroid and Other Endocrine Glands	C73, C74, C75
Urinary Tract (Kidney, Ureter, Bladder)	C64, C65, C66, C67, C68
Ill-Defined, Other Secondary, and Unspecified Sites	C76, C77, C78, C79, C80
Independent (Primary) Multiple Sites	C97

© 2025 Potter AL et al. *JAMA Network Open*.

eReferences.

1. National Center for Health Statistics. Data access—vital statistics online (mortality multiple cause files). https://www.cdc.gov/nchs/data_access/vitalstatsonline.htm#Mortality_Multiple.
2. Martin, J., Hamilton, B., Osterman, M., and Driscoll, A. (2021). Births: Final data for 2019. National Vital Statistics Reports; vol 70 no 2. https://dx.doi.org/10.15620/cdc:100472.
3. Osterman, M., Hamilton, B., Martin, J., Driscoll, A., and Valenzuela, C. (2022). Births: Final data for 2020. National Vital Statistics Reports; vol70 no 17. https://dx.doi.org/10.15620/cdc:112078.
4. Human Fertility Collection. Max Planck Institute for Demographic Research (Germany) and Vienna Institute of Demography (Austria). Available at https://www.fertilitydata.org (data downloaded on 25 March 2024).
5. Schlüter B-S, Alburez-Gutierrez D, Bibbins-Domingo K, Alexander MJ, Kiang MV. Youth Experiencing Parental Death Due to Drug Poisoning and Firearm Violence in the US, 1999-2020. *JAMA*. 2024;331(20):1741-1747. doi:10.1001/jama.2024.8391
6. Centers for Disease Control and Prevention (2021). US census populations with bridged race categories. https://www.cdc.gov/nchs/nvss/bridged_race.htm.
7. Caswell H. The formal demography of kinship

   A matrix formulation. *Demographic Research*. 2019;41:679-712.
8. Caswell H, Song X. The formal demography of kinship III: Kinship dynamics with time-varying demographic rates. *bioRxiv*. 2021;
9. Caswell H. The formal demography of kinship IV: Two-sex models and their approximations. *Demographic Research*. 09/07 2022;47:359-396. doi:10.4054/DemRes.2022.47.130
10. Harris ID, Fronczak C, Roth L, Meacham RB. Fertility and the aging male. *Rev Urol*. 2011;13(4):e184-90.

© 2025 Potter AL et al. *JAMA Network Open*.
