# Supplementary material for: Youths Experiencing Parental Death Due to Cancer
Source: JAMA Netw Open. 2025 Jul 7;8(7):e2519106. doi: 10.1001/jamanetworkopen.2025.19106 (PMC12235492; doi:10.1001/jamanetworkopen.2025.19106)
Supplement: Supplement 2. — Data Sharing Statement [file jamanetwopen-e2519106-s002.pdf]

Data Sharing Statement

Potter. Youths Experiencing Parental Death Due to Cancer. *JAMA Netw Open*. Published July 07, 2025. doi:10.1001/jamanetworkopen.2025.19106

Data
Data available: Yes
Data types: Deidentified participant data
How to access data: Data will be made available on our Github repository or an Open Science Framework (OSF) repository as appropriate. In addition, all data are publicly available and our code will be made publicly available upon publication such that the analysis can be replicated using just publicly accessible data.
https://github.com/mkiang/parental_deaths_cancer
When available: With publication

Supporting Documents
Document types: Statistical/analytic code
How to access documents: https://github.com/mkiang/parental_deaths_cancer
When available: With publication

Additional Information
Who can access the data: Data will be publicly available on a linked OSF repository or via our Github repository: https://github.com/mkiang/parental_deaths_cancer
Types of analyses: Any purpose.
Mechanisms of data availability: Publicly available.
Any additional restrictions: Only restrictions are those that the NCHS puts in place (e.g., no reidentification).
